# The mutational pattern of homologous recombination-related (HRR) genes in Chinese colon cancer and its relevance to immunotherapy responses

**DOI:** 10.18632/aging.202267

**Published:** 2020-12-09

**Authors:** Pei Zhou, Xueying Wu, Huan Chen, Ying Hu, Henghui Zhang, Lijia Wu, Ying Yang, Beibei Mao, Huaqing Wang

**Affiliations:** 1 Department of Medical Oncology, Tianjin Union Medical Center, The Affiliated Hospital of Nankai University, Tianjin, China; 2 Genecast Precision Medicine Technology Institute, Beijing, China; 3 Center of Integrative Medicine, Beijing Ditan Hospital, Capital Medical University, Beijing, China; 4 Institute of Infectious Diseases, Beijing Ditan Hospital, Capital Medical University, Beijing, China

**Keywords:** colon cancer, biomarker, homologous recombination deficiency, immunotherapy, microsatellite stable

## Abstract

Background: Microsatellite-stable (MSS) colon adenocarcinoma (COAD) patients are not sensitive to immune checkpoint inhibitors. Here, we focused on analyzing the relationship between homologous recombination repair (HRR)-related gene mutations and clinical immunotherapy responses in MSS COAD.

Methods: The mutational landscape was profiled in a cohort of 406 Chinese COAD patients via next-generation sequencing (NGS). Correlations between HRR gene mutations and tumor immunity or clinical outcomes in two COAD genomic datasets were analyzed via bioinformatics.

Results: In the Chinese cohort, seventy (17%) patients exhibited genomic alterations in HRR genes; *ATM* (9%), *BRCA2* (4%), *ATR* (3%), *RAD50* (3%) and *BRIP1* (3%) were the most frequently mutated. In the MSK-IMPACT COAD cohort (immune checkpoint inhibitor-treated), HRR-mut patients (n=34) survived longer than HRR-wt patients (n=50) (log-rank *P* < 0.01). Based on the TCGA MSS COAD cohort, HRR gene mutations increased immune activities, such as infiltration of cytotoxic cells (*P* < 0.05) and exhausted CD8+ T cells (*P* < 0.01), and increased the IFN-γ scores (*P* < 0.05). The results differed in MSI-H COAD patients (all *P* > 0.05).

Conclusion: HRR gene mutations significantly increased immune activities in MSS COAD patients, implying the feasibility of the HRR-mut status as an immunotherapy response predictor in MSS COAD.

## INTRODUCTION

Colorectal cancers (CRCs), including colon cancer (CC) and rectal cancer (RC), are the most commonly diagnosed cancers of the alimentary tract epithelium, and >95% of CRCs are colon adenocarcinoma (COAD) and rectal adenocarcinoma (READ) [1, 2]. Surgery combined with chemotherapy or radiation therapy is the main therapeutic strategy for CRC, but the treatment options for unresectable, locally advanced CRC remain limited [3].

Currently, attention regarding the use of immune checkpoint inhibitors (ICIs) to treat CRC is increasing, and identifying biomarkers that predict the response to ICIs is thus critical for achieving the full potential of these immunotherapies. Unfortunately, the DNA mismatch repair (MMR) status is the only well-established biomarker in the National Comprehensive Cancer Network (NCCN) guidelines for CRC [4]. Patients with CRC exhibiting a microsatellite instability-high (MSI-H) status or mismatch repair deficiency (dMMR) have been reported to be sensitive to pembrolizumab [5, 6]. However, the vast majority of CRC patients (85%) have microsatellite instability-low (MSI-L) or microsatellite-stable (MSS) tumors, and these populations are historically not responsive to ICIs. However, preliminary data on the combination of monalizumab and durvalumab in a cohort of patients with MSS CRC are encouraging (NCT02671435) [7]. Therefore, potential predictive therapeutic biomarkers are urgently needed to increase the benefit of ICIs for patients with MSS CRC. Furthermore, according to epidemiological statistics, colon cancer and rectal cancer exhibit significantly different progression mechanisms and etiologies despite collectively being called CRC [8]; thus, they may need to be studied separately.

Several studies on gynecological cancers have demonstrated that homologous recombination deficiency (HRD) can modify the tumor immune microenvironment by increasing the number of tumor-infiltrating lymphocytes (TILs) [9], indicating that HRD might be a biomarker for the immunotherapy response. Although HRD was initially defined as germline *BRCA1* or *BRCA2* mutation [10], as next-generation sequencing (NGS) was developed, several studies in gynecological cancer have suggested that patients with somatic mutations in components of the homologous recombination repair (HRR) pathway are also likely to have an HRD phenotype [11–13]. However, comprehensive evaluations of HRR in COAD have not been conducted, and the association between HRR gene mutations and the immunotherapy response in COAD has not been investigated.

In this study, we first used a large Chinese COAD cohort of 406 patients to illustrate the HRR somatic mutation profiles and related molecular characteristics (tumor mutation burden (TMB) and MSI data). Furthermore, to analyze the COAD cohort from The Cancer Genome Atlas (TCGA), we compared the immune characteristics between the HRR-mut and HRR-wt groups of all patients, MSI-H patients, and MSS patients to explore the feasibility of the HR-mut status as an immunotherapy biomarker in COAD, especially MSS COAD.

## RESULTS

### Mutational landscape of HRR genes in Chinese COAD patients

**Figure 1:**
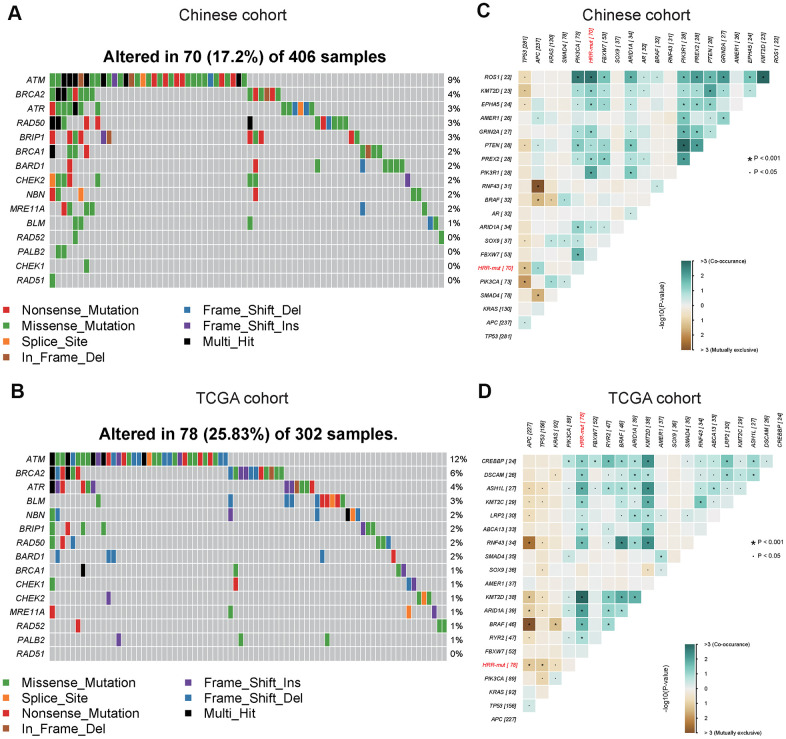
**Mutational landscape and genomic patterns of HRR genes in COAD.** (**A**, **B**) Mutational landscape of HR genes in the Chinese cohort (**A**) and TCGA cohort (**B**). The columns and rows represent patients and genes, respectively. The patients are sorted in decreasing order by the number of patients in whom a gene is mutated. The right panel indicates the frequency of gene mutations. Mutation types are indicated by different colors. Gray denotes an absence of mutations. (**C**, **D**) Co-occurring and exclusive somatic mutations in the Chinese cohort (**C**) and TCGA cohort (**D**). *P* values were calculated using Fisher’s exact test. All somatic mutated HRR genes were masked as HRR-mut. These figures were generated with the “somaticInteractions” functions in the maftools package.

To better understand the genomic alteration profile of Chinese COAD patients, we performed NGS on a panel of 543 cancer-related genes to search for somatic mutations. The three most frequently mutated genes were *TP53*, *APC* and *KRAS* in COAD, and the mutation frequency of *TP53* in the Chinese cohort (70%) was higher than that in the TCGA cohort (52%) (Supplementary Figure 1). Similar mutational patterns in HRR genes were observed in both cohorts; *ATM*, *BRCA2*, and *ATR* were among the most frequently mutated HRR genes. Overall, the mutation frequency of HRR genes in the Chinese cohort (70/406, 17%) was lower than that in the TCGA cohort (78/302, 26%) (Figure 1A, 1B). Further analysis of genetic interactions revealed that the HRR-mut status (all somatic mutations in HRR genes were masked as HRR-mut) was co-occurrent with alterations in *KMT2D* but exclusive to alterations in *TP53* in both cohorts (Figure 1C, 1D).

Subjects with somatic mutations in these core HR pathway genes (see “Materials and Methods”) were included in the HRR-mut group (n=70, Chinese cohort; n=78, TCGA cohort) in subsequent analyses.

### Mutations in HRR genes are associated with the TMB and the MSI status

**Figure 2:**
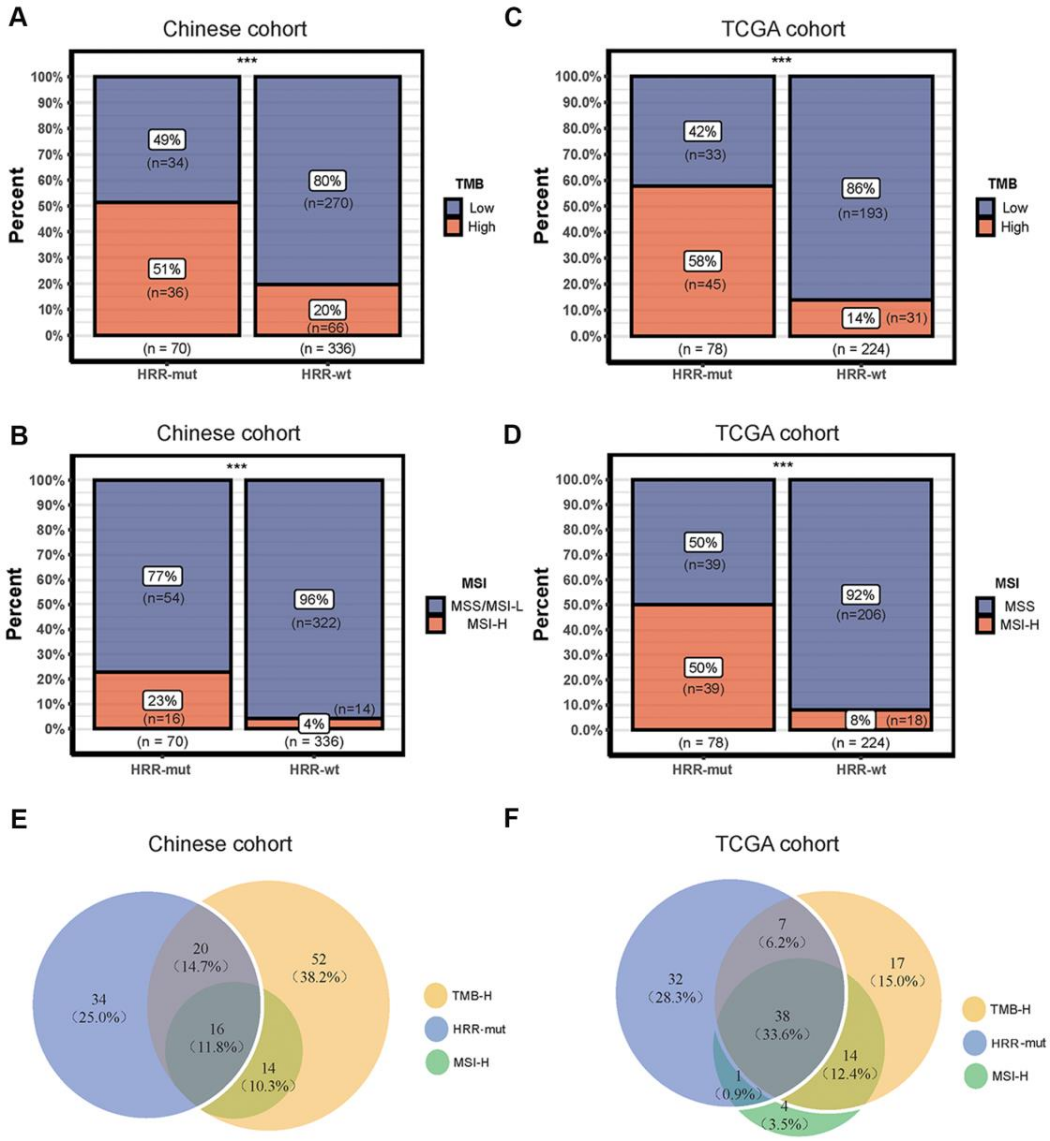
**Associations among mutations in HRR genes, TMB and the MSI status.** (**A**–**C**) Bar plots showing the percentage of TMB-high patients in the HRR-mut group compared with the HRR-wt group in the Chinese (**A**) and TCGA (**C**) cohorts. (**B**–**D**) Bar plots showing the percentage of MSI-H patients in the HRR-mut group compared with the HRR-wt group in the Chinese (**B**) and TCGA (**D**) cohorts. Comparisons between the groups were performed with Fisher’s exact test (* *P* < 0.05; ** *P* < 0.01; *** *P* < 0.001; ns, *P* > 0.05). (**E**, **F**) Venn diagrams illustrating the overlap between patients with HRR-mut, TMB-H, and MSI-H COAD in the Chinese (**E**) and TCGA (**F**) cohorts.

Although the TMB is not currently used as an immunotherapy biomarker in CRC, it has been suggested to play an important role in guiding the sequence and/or combination of ICIs in the treatment of MSI-H mCRC [22]. Therefore, we next analyzed the associations among MSI-H status, TMB, and HRD. In the Chinese cohort, the HRR-mut COAD group had a significantly higher TMB and incidence of MSI-H status than the HRR-wt COAD group (TMB value above the top 25^th^ percentile was defined as TMB-H; see “Materials and Methods”; Fisher’s exact test, *P* < 0.001; Figure 2A, 2B). Similar results were also obtained for the TCGA cohort (Figure 2C, 2D). Venn diagrams were used to visualize the relationships among high TMB, MSI-H status, and HRR-mut status for patients with COAD in our cohort (Figure 2E) and in the TCGA cohort (Figure 2F). Almost all patients with MSI-H tumors also had a high TMB, consistent with previously reported results from Foundation Medicine [23].

### HRR-mut is associated with elevated immune activity in COAD

**Figure 3:**
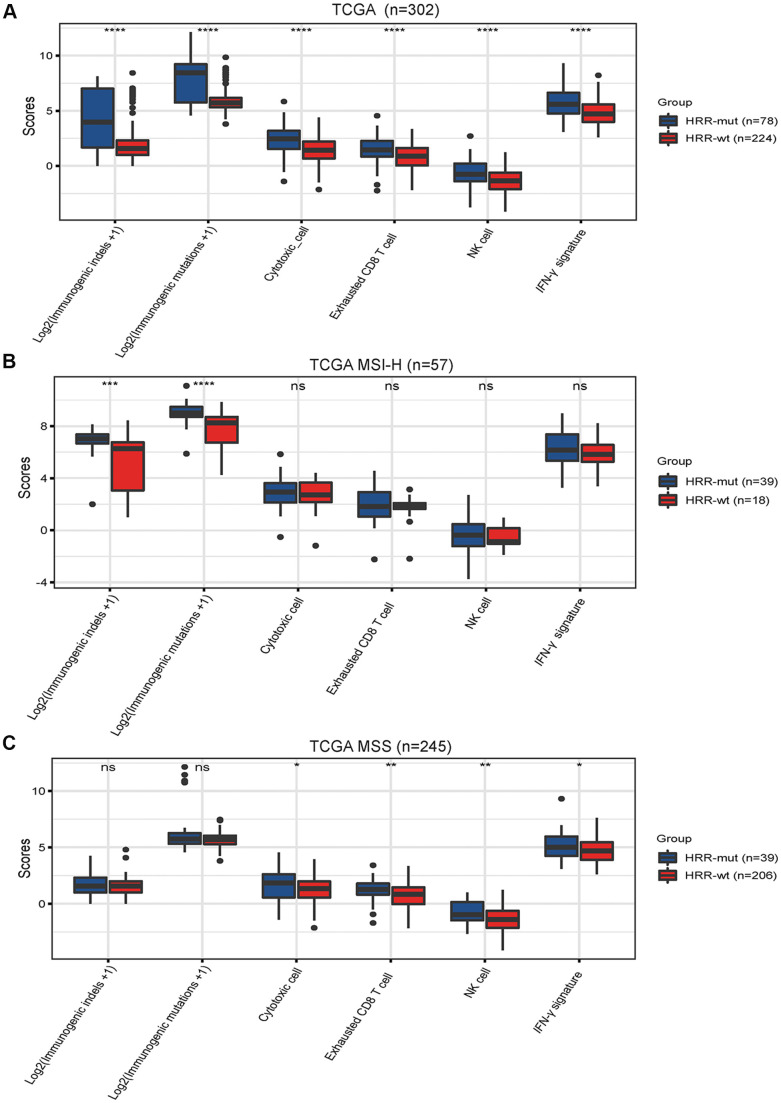
**Mutations in HRR genes are associated with tumor immunogenicity and immune activity.** Box plots showing the scores of immunogenic mutations, immunogenic indels, cytotoxic cells, exhausted CD8+ T cells, NK cells and IFN-γ signatures in the TCGA (**A**), TCGA MSI-H (**B**) AND TCGA MSS (**C**) cohorts. The scores for immunogenic mutations and indels are shown in log2-transformed format. *P* values were calculated with the Mann–Whitney U test; the box shows the upper and lower quartiles (* *P* < 0.05; ** *P* < 0.01; *** *P* < 0.001; ns, *P* > 0.05).

While immune activity is positively correlated with the TMB, very few of these mutations can generate mutant antigens that have high affinity for major histocompatibility complexes (MHCs) and thus be recognized by T cells [24, 25]. Neoantigens derived from immunogenic mutations or immunogenic indels can reportedly elicit potent immune activity and indicate a better response to immunotherapy in various cancer types [26]. Unsurprisingly, the neoantigen burden was significantly higher in the HRR-mut COAD group than in the HRR-wt COAD group (Figure 3A, *P* <0.001). Next, we investigated whether immune-related signatures correlating with the response to ICIs are also altered in patients with HRR-wt COAD by analyzing the IFN-γ response signatures [20] and TIL scores [19] in gene expression profiles using RNA data from the GDC Data Portal (https://portal.gdc.cancer.gov/, see “Materials and Methods”). The levels of these immune signatures were higher in patients with HRR-mut COAD than in patients with HRR-wt COAD (Figure 3A, all *P* <0.001).

Next, we performed similar analyses for MSI-H COAD and MSS COAD. In MSI-H COAD, mutations in HRR genes significantly affected the genomic profiles (immunogenic mutations or indels) but did not affect the transcriptome signatures (IFN-γ response signatures and TIL scores) (Figure 3B). Intriguingly, completely opposite results were observed in the MSS COAD group (Figure 3C). These data suggest that the HRR-mut status contributes to enhanced immune activity but functions differently in MSI-H COAD and MSS COAD.

### HRR-mut is associated with a favorable therapeutic response to ICIs

**Figure 4:**
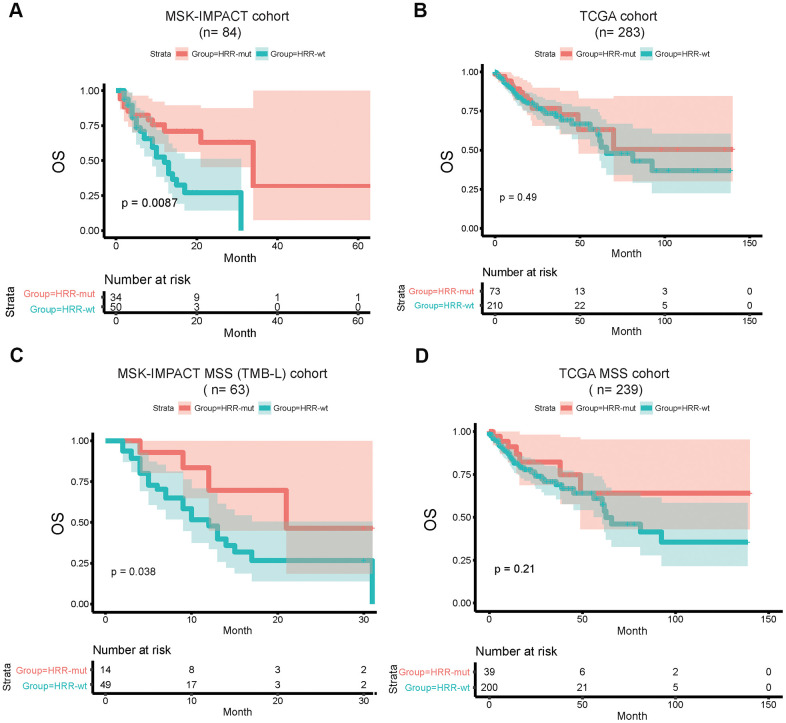
**HRR-mut status is associated with a favorable therapeutic response to ICIs.** Kaplan–Meier survival curves showing the OS times of patients stratified by HRR-mut/wt status in the MSK-IMPACT (**A**) and TCGA (**B**) cohorts. Kaplan–Meier survival curves showing the OS times of patients stratified by HRR-mut/wt status in the MSK-IMPACT MSS (TMB-L) (**C**) and TCGA MSS (**D**) cohorts. *P* values were calculated with the log-rank test.

Finally, we explored the correlation between the HRR-mut status and the response to ICIs using an ICI-treated cohort (MSK-IMPACT cohort) [21] comprising 84 patients. The TCGA COAD cohort (non-ICI-treated) was used as a comparison cohort. In the MSK-IMPACT cohort, patients with HRR-mut COAD (n=34) had significantly better OS than patients with HRR-wt COAD (n=50) (log-rank *P* < 0.05; Figure 4A). Due to the lack of MSI status information in the MSK-IMPACT cohort, we elected to use the TMB-L population as a representative MSS subgroup for the survival analysis and found that the HRR-mut status was also significantly associated with a better prognosis for patients with TMB-L (MSS) COAD than for those with HRR-wt COAD (log-rank *P* < 0.05; Figure 4C). However, the correlation between HRR-mut status and OS was not significant in patients in the TCGA cohort with COAD/MSS COAD treated with traditional therapy (log-rank *P* > 0.05; Figure 4B–4D).

## DISCUSSION

In this study, we defined an HRR-mut group with a potential HRD phenotype and performed mutational landscape analysis of 543 cancer-related genes in a large Chinese cohort comprising only COAD patients. Our data indicated that 17% (Figure 1A) of Chinese COAD patients may benefit from various treatments associated with the HR pathway. In the TCGA COAD cohort, the mutation frequencies of HRR genes in Chinese patients were relatively low compared with those in Western patients. Therefore, HRR gene mutations may have occurred mutually exclusively with *TP53* alterations and concurrently with KMT2D in these COAD patients (Figure 1C, 1D); moreover, *TP53* was mutated more frequently in Chinese patients than in Western patients (Supplementary Figure 1).

Recent research on several gynecological cancers has shown a strong relationship between HRD and increased immune cell infiltration [9]. These studies lay the groundwork for future research on the potential use of HRD as an immunotherapy biomarker. The approval of PD-1 inhibitors as first-line treatments has led to encouraging clinical outcomes in advanced COAD [27]. However, evidence for an association between commonly used predictive markers, such as PD-L1 expression, and the response to ICIs in COAD is lacking [28]. The current study showed that the HRR-mut status was generally associated with elevated immune activity and tumor immunogenicity in all COAD cases. Moreover, patients with HRR-mut COAD had a significantly higher OS rate than patients with HRR-wt COAD after ICI treatment. Based on the above results, we speculate that somatic mutations in HRR genes are potential biomarkers for the response to ICIs in COAD.

Interestingly, in patients with MSS COAD, all transcriptome profiles that predict the clinical response to PD-1 blockade were increased in the HRR-mut group compared with the HRR-wt group. To date, the dMMR/MSI status remains the only clear marker for benefit from PD-1 blockade therapy in patients with intestinal cancer. However, according to a new report, first-line durvalumab combined with monalizumab showed a manageable safety profile and preliminary activity in patients with advanced/metastatic MSS CRC in a phase I/II trial (NCT02671435) [7]. The above data strongly indicate that novel immunotherapy biomarkers for MSS COAD will be identified. Unfortunately, due to the lack of MSI status information in the MSK-IMPACT cohort, we could use only the TMB-L population as a representative MSS subgroup to analyze the effect of the HRR-mut status on the clinical efficacy of ICIs. Although the cutoff value used to stratify the TMB-L population was lower in our study than in the MSK-IMPACT study [21] (75% vs. 80%), we cannot guarantee that the TMB-L group was composed entirely of MSS COAD patients; thus, these findings should be interpreted with caution. However, the present study suggests the potential of using the HRD status as a predictive biomarker for the response to ICIs in patients with MSS COAD.

In addition, *ATM* was the most frequently mutated HRR gene in COAD patients. Recently, ATM was reported to be a predictive marker of the response to treatment with epidermal growth factor receptor (EGFR)-targeted therapies, as aggregated mutations in ATM are correlated with treatment unresponsiveness [29]. Therefore, detecting somatic mutations in HRR genes might provide guidance for various drug treatment options, not just poly(ADP ribose) polymerase inhibitors (PARPi) and ICIs.

### Limitations

This study has several limitations. First, due to data restrictions, we did not have transcriptome data for the Chinese cohort and thus could not validate the findings obtained with the TCGA cohort. Second, an ICI-treated MSS COAD cohort is needed to verify the conclusions regarding survival. Therefore, further studies are warranted.

## CONCLUSION

In summary, our data suggest that detecting somatic mutations in HRR genes might increase the proportion of patients with COAD—especially MSS COAD—who might benefit from immune checkpoint blockade therapy.

## MATERIALS AND METHODS

### Homologous recombination (HR) status definition

The HRD phenotype has been defined as the presence of a non-silent somatic mutation in *RAD51, CHEK1, PALB2, RAD52, BLM, MRE11A, NBN, CHEK2, BARD1, BRCA1, BRIP1, RAD50, ATR, BRCA2,* or *ATM*, which have been reported to be core genes in the HRR pathway [14]. Details are described in Supplementary Table 1.

### Patient information and sample collection

To analyze the prevalence of HR gene mutations in COAD, we collected genomic data for 708 patients diagnosed with colon cancer from 2 cohorts: (1) a Chinese cohort comprising 406 Chinese patients (provided by Tianjin Union Medical Center, the Affiliated Hospital of Nankai University; all patients provided written informed consent); and (2) a TCGA cohort, comprising 302 patients (with TMB and MSI data). The single nucleotide variant (SNV) data for the TCGA cohort were obtained from the GDC Data Portal (https://portal.gdc.cancer.gov/). The inclusion/exclusion criteria for the samples were as follows: 1) only tumor samples were included, 2) the primary site was the colon, and 3) all silent mutations were ignored.

This study was approved by the Institutional Review Board of Tianjin Union Medical Center.

### DNA extraction and sequencing

Formalin-fixed, paraffin-embedded (FFPE) tissue specimens of the primary tumor from each patient were collected for analysis. The black PREP FFPE DNA Kit (Analytik Jena, Germany) was used to isolate DNA from the FFPE tissue specimens. Whole blood samples were centrifuged for 10 minutes (1,600 g) at room temperature to isolate lymphocytes, and a Tiangen Whole Blood DNA Kit (Tiangen, Beijing, China) was used to extract DNA from peripheral blood lymphocytes according to the manufacturer's instructions. Genomic DNA was sheared into 150-200 bp fragments with a Covaris M220 focused ultrasonicator (Covaris, Massachusetts, USA), and a DNA fragment library was constructed using a KAPA HTP Library Preparation Kit for the Illumina platform (KAPA Biosystems, Massachusetts, USA) according to the manufacturer's instructions. The DNA library was captured with a 543-gene plate designed based on the NimbleGen SeqCap EZ library (Roche, Wisconsin, USA), which includes key tumor-related genes. Captured samples were subjected to paired-end sequencing on the Illumina HiSeq X-Ten (cohort 1) or NovaSeq 6000 (cohort 2) platform.

### Variant calling

Somatic cell SNVs in blood samples were identified by VarScan2 (v2.4.2) with the following parameters: (1) number of mutant allele reads > 2; (2) normal read coverage > 50 and tumor read coverage > 100; (3) mutated allele frequency > 2%; (4) nonsynonymous SNVs and insertion/deletions (indels) (5) located in exonic regions; and (6) an allele frequency of < 0.5% in the exac03 database.

### Analysis of the TMB and MSI status in the Chinese cohort

The TMB (mutations per megabase (Mb) of DNA) was extrapolated using sequencing data from the panel of 543 cancer-related genes and determined by analyzing the number of somatic mutations per megabase. The top 25^th^ percentile of the TMB value was used as the cutoff value to define tumors with a high mutation burden (TMB-H tumors) in this study.

Tumor DNA samples were subjected to NGS using the cancer gene-targeted panel. Seventy target microsatellite loci were examined and compared with those in genomic data from healthy people in the Chinese database. The number of microsatellite loci altered by somatic insertions or deletions was determined for each patient sample. If the ratio of unstable loci to passing loci was equal to or higher than 0.3, the MSI status of the sample was defined as MSI-H; meanwhile, if the ratio of unstable loci to passing loci was less than 0.3, the MSI status of the sample was defined as MSI-L/MSS.

### Associations of HRD with the TMB, the MSI status, the neoantigen burden, and aneuploidy in the TCGA cohort

The TMB, MSI, immunogenic somatic mutation, copy number variation (CNV) and loss of heterozygosity (LOH) data were downloaded from published studies [15–18] with the TCGA cohort.

### Immune-related signature analysis

The gene sets used for TILs (cytotoxic cells, exhausted CD8+ T cells and natural killer (NK) cells) and the IFN-γ signature were the same as those used in previous studies [19, 20] (Supplementary Table 2, Supplementary Table 3). TCGA transcriptome profiling data were obtained from the GDC Data Portal (https://portal.gdc.cancer.gov/). The expression of each target gene was normalized by transcripts per kilobase million (TPM) normalization, and the immune signatures were assessed as the geometric mean of the gene expression levels in log2(TPM+1) format.

### Association between HRD and the survival outcome

We compared the overall survival (OS) between the HRR-mut and HRR-wt COAD groups in an ICI-treated cohort (MSK-IMPACT cohort) [21]. Kaplan–Meier survival curves were used to visualize the survival differences, and the log-rank test was used to evaluate the significance of differences in the survival time. We performed survival analyses using the R function “survfit” in the “survival” package.

### Statistical analysis

All statistical analyses were performed using R version 3.6.1 software (Institute for Statistics and Mathematics, Vienna, Austria; https://www.r-project.org). Fisher’s exact test was applied for comparisons between two categorical variables, and the Mann–Whitney U test was used for comparisons between two continuous variables. A survival analysis was performed using a Kaplan–Meier survival plot, and log-rank *P* values were calculated. All differences with *P* < 0.05 were considered statistically significant.

## Supplementary Material

Supplementary Figure 1

Supplementary Tables
