# Supplementary material for: The mutational pattern of homologous recombination-related (HRR) genes in Chinese colon cancer and its relevance to immunotherapy responses
Source: Aging (Albany NY). 2020 Dec 9;13(2):2365–78. doi: 10.18632/aging.202267 (PMC7880324; doi:10.18632/aging.202267)
Supplement: Supplementary Figure 1 [file aging-13-202267-s001.pdf]

SUPPLEMENTARY FIGURE

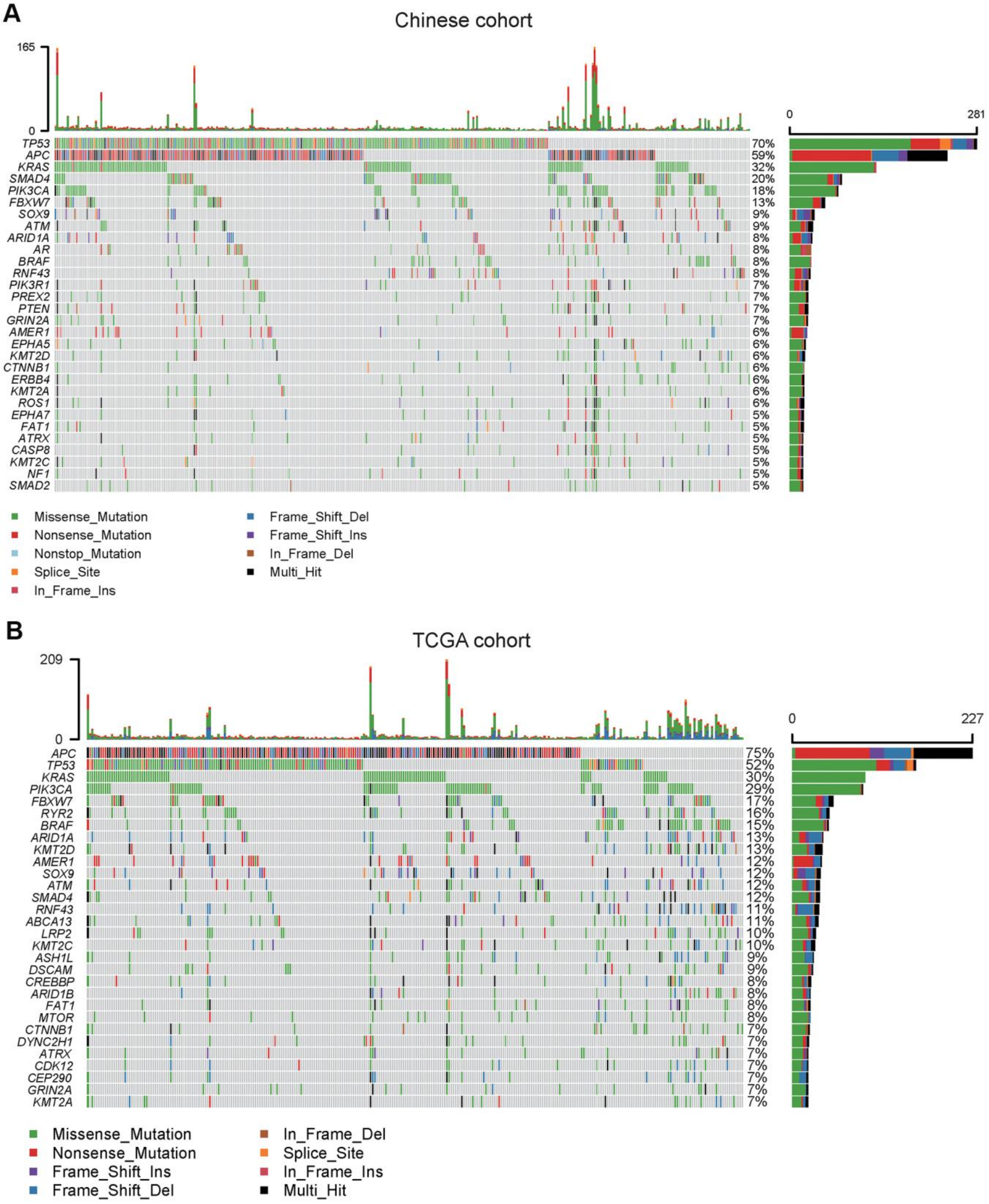

Supplementary Figure 1. Mutational landscape in COAD patients. Mutational landscape of the 30 most frequently mutated genes in the Chinese cohort (**A**) and TCGA cohort (**B**). The columns and rows represent patients and genes, respectively, and are sorted in decreasing order by the number of patients in whom a gene is mutated. The right panel indicates the frequency of gene mutations. Mutation types are differentiated by colors.
