# Supplementary material for: The mutational pattern of homologous recombination-related (HRR) genes in Chinese colon cancer and its relevance to immunotherapy responses
Source: Aging (Albany NY). 2020 Dec 9;13(2):2365–78. doi: 10.18632/aging.202267 (PMC7880324; doi:10.18632/aging.202267)
Supplement: Supplementary Tables [file aging-13-202267-s002.pdf]

SUPPLEMENTARY TABLES

Supplementary Table 1. Annotation of 15 core members of the HR pathway.

Entrez gene ID	Gene symbol	Gene description	Approved name	HGNC ID	Location
4361	MRE11A	MRE11 homolog A, double strand break repair nuclease	MRE11 homolog, double strand break repair nuclease	HGNC:7230	11q21
4683	NBN	Nibrin	Nibrin	HGNC:7652	8q21.3
10111	RAD50	RAD50 double strand break repair protein	RAD50 double strand break repair protein	HGNC:9816	5q31.1
580	BARD1	BRCA1 associated RING domain 1	BRCA1 associated RING domain 1	HGNC:952	2q35
641	BLM	Bloom syndrome, RecQ helicase-like	Bloom syndrome RecQ like helicase	HGNC:1058	15q26.1
672	BRCA1	breast cancer 1, early onset	BRCA1, DNA repair associated	HGNC:1100	17q21.31
675	BRCA2	Breast cancer 2, early onset	BRCA2, DNA repair associated	HGNC:1101	13q13.1
83990	BRIP1	BRCA1 interacting protein C-terminal helicase 1	BRCA1 interacting protein C-terminal helicase 1	HGNC:20473	17q23.2
79728	PALB2	Partner and localizer of BRCA2	Partner and localizer of BRCA2	HGNC:26144	16p12.2
5888	RAD51	RAD51 recombinase	RAD51 recombinase	HGNC:9817	15q15.1
5893	RAD52	RAD52 homolog, DNA repair protein	RAD52 homolog, DNA repair protein	HGNC:9824	12p13.33
472	ATM	ATM serine/threonine kinase	ATM serine/threonine kinase	HGNC:795	11q22.3
545	ATR	ATR serine/threonine kinase	ATR serine/threonine kinase	HGNC:882	3q23
1111	CHEK1	Checkpoint kinase 1	Checkpoint kinase 1	HGNC:1925	11q24.2
11200	CHEK2	Checkpoint kinase 2	Checkpoint kinase 2	HGNC:16627	22q12.1

Supplementary Table 2. IFN-γ signature gene set.

Gene
IDO1
CXCL10
CXCL9
HLA-DRA
STAT1
IFNG

Supplementary Table 3. TIL score gene sets.

Cytotoxic cells	Exhausted CD8 cells	NK cells
CTSW	CD244	NCR1
GNLY	EOMES	XCL2
GZMA	LAG3	XCL1
GZMB	PTGER4	
GZMH		
KLRB1		
KLRD1		
KLRK1		
PRF1		
NKG7		
